# Multiple timescales of learning indicated by changes in evidence-accumulation processes during perceptual decision-making

**DOI:** 10.1038/s41539-023-00168-9

**Published:** 2023-06-08

**Authors:** Aaron Cochrane, Chris R. Sims, Vikranth R. Bejjanki, C. Shawn Green, Daphne Bavelier

**Affiliations:** 1 University of Geneva, Geneva, Switzerland; 2 Campus Biotech, Geneva, Switzerland; 3 Brown University, Providence, RI USA; 4 Rensselaer Polytechnic Institute, Troy, NY USA; 5 Hamilton College, Clinton, NY USA; 6 University of Wisconsin—Madison, Madison, WI USA

**Keywords:** Human behaviour, Decision making, Motion detection

## Abstract

Evidence accumulation models have enabled strong advances in our understanding of decision-making, yet their application to examining learning has not been common. Using data from participants completing a dynamic random dot-motion direction discrimination task across four days, we characterized alterations in two components of perceptual decision-making (Drift Diffusion Model drift rate and response boundary). Continuous-time learning models were applied to characterize trajectories of performance change, with different models allowing for varying dynamics. The best-fitting model included drift rate changing as a continuous, exponential function of cumulative trial number. In contrast, response boundary changed within each daily session, but in an independent manner across daily sessions. Our results highlight two different processes underlying the pattern of behavior observed across the entire learning trajectory, one involving a continuous tuning of perceptual sensitivity, and another more variable process describing participants’ threshold of when enough evidence is present to act.

## Introduction

Learning occurs in nearly every behavior that humans perform, from complex cognitive or motor tasks to basic perceptual discriminations. Understanding the processes responsible for learning therefore has implications for most human actions in contexts ranging from education to rehabilitation^1,2^. Surprisingly, then, research on learning processes often tends to be limited in several ways. First, it is quite common for research on learning to focus exclusively on *either* the accuracy or identity of choices^3,4^ or the time taken to make choices (i.e., response time or RT^5,6^). This is despite a vast amount of research showing that richer inferences regarding the processes at hand become possible when considering *both* the speed of decisions and their accuracy in combination^7–10^. Second, in the limited set of cases where both RT and accuracy have been considered in learning studies, modelling has nearly always involved substantial aggregation across participants and/or learning trials. Often, a separate stationary model is fit to all of the trials from each individual learning session or “block” of trials and then learning is examined through differences in the model parameters from session to session (or block to block). Such aggregation remains common despite research showing that it may be both theoretically and empirically ill-advised, as it can lead to missed or erroneous inferences about the underlying learning processes^11–13^. Below we briefly review (A) modelling approaches to linking RT and accuracy and their previous use in the assessment of learning in the perceptual domain and (B) continuous-time and individual-participant approaches to assessing learning in the perceptual domain, in order to motivate the need to combine these two approaches in a single framework. This combined approach allows us to address key questions regarding the way in which specific aspects of the perceptual decision-making process (e.g., how quickly perceptual evidence accumulates, how much evidence is needed before triggering a response) change through time in a multi-session perceptual learning study.

**Fig. 1:**
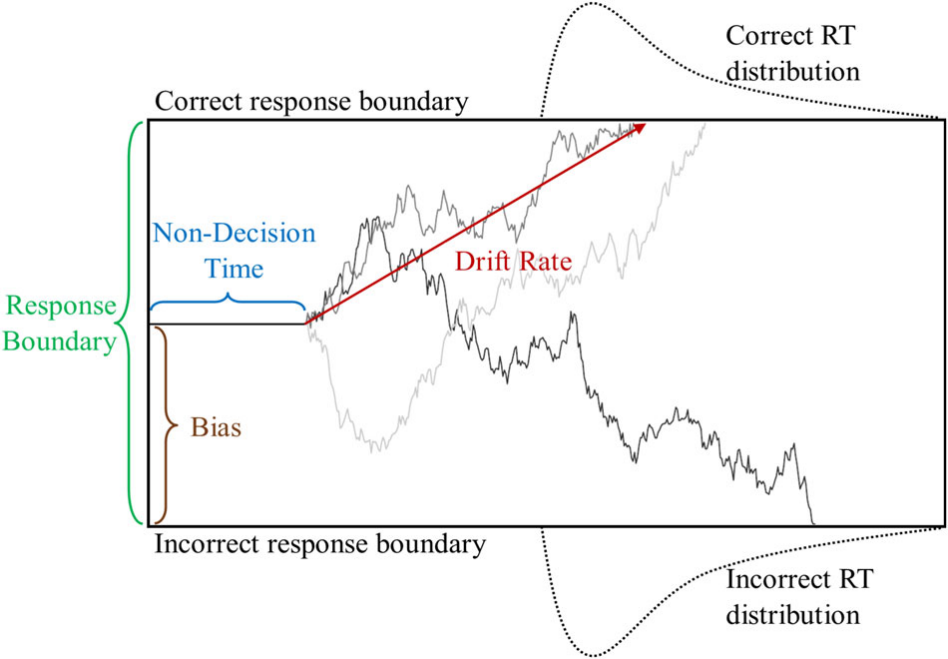
Drift diffusion model. The decision-making process is defined by a noisy accumulation of evidence (three examples indicated by grey lines) with some mean value, or Drift Rate, to either a lower or an upper bound, also termed Response Boundary. Evidence accumulation begins at some Bias point (typically expressed as a proportion of the Response Boundary), and an additive Non-Decision Time parameter accounts for aspects of the RT distributions that are not attributable to the evidence-accumulation process.

The most prominent model linking RT and discrete choice (e.g., accuracy) conceptualizes behaviors as resulting from the noisy accumulation of evidence until a decision boundary is reached^7,9,10,14^. For example, consider a task where participants view a field of moving dots (see Supplementary Fig. 1). On each trial, 15% of the dots move coherently either to the left or to the right, while the rest of the dots move randomly. The participants’ task is to, as quickly and accurately as possible, indicate whether the coherently moving dots are moving to the left or to the right. Under an accumulator modeling approach, the perceptual system continuously accrues evidence in favor of the respective alternatives (left or right), until the amount of evidence in favor of one of the alternatives reaches some boundary, at which point the given decision is made. This model is formally defined by a bounded Wiener diffusion process^9,10^. The resulting Drift Diffusion Model (DDM) is characterized by four parameters (see Fig. 1). In two alternative forced choices, evidence begins accumulation at a *bias* point and proceeds with a constant noise and a *drift rate* (DR) until it reaches a boundary, with the distance between the boundaries being defined by a *response boundary* (RB) parameter. A last parameter is an additive value to the RT that is theoretically independent of the evidence-accumulation process (*non-decision time*; NDT). Estimating these parameters using hierarchical Bayesian methods has become increasingly common, which formulates DDM parameter estimation as generalized mixed-effects regression fitted to the joint RT and accuracy distributions^8,15,16^. Here we implement similar methods using Stan^17–19^.

DDM parameters are theoretically associated with psychological components of decision-making processes (noting, however, that there is frequent debate regarding the empirical isomorphism between DDM parameters and processes^7,20^, but see ref. ^21^^,^). Sensitivity, such as would be associated with better or worse perceptual abilities, is generally associated with DR. Response caution or a “speed-accuracy tradeoff” is generally associated with RB. In the present study, only DR and RB were modeled as changing with time, with bias and NDT being held constant over time within participants. While it is the case that bias and NDT parameters have been shown to change when strategies are experimentally manipulated^20,22^ and NDT variability has been seen to be reduced during some perceptual learning tasks^23^, there are also known trade-offs in estimation methods leading to inferred differences in NDT when the true distributional differences are due to other parameters, such as DR and RB^14^. In light of this, bias and NDT were estimated for each participant as a constant, with only DR and RB considered as time varying according to an exponential learning function ${\boldsymbol{asymptote}}{\boldsymbol{+}}{\boldsymbol{(}}{\boldsymbol{start}}{\boldsymbol{-}}{\boldsymbol{asymptote}}{\boldsymbol{)}}{\boldsymbol{* }}{{\boldsymbol{2}}}^{{\boldsymbol{trialNumber}}{\boldsymbol{/}}{\boldsymbol{rate}}}$ see Methods and Supplementary Note; ^3,5,24^.

Given the putative links between DDM parameters and psychological components of the speeded decision-making process, one may expect such models to be prominent across most related sub-domains of research. However, this is not necessarily the case. For instance, perceptual learning^25^ is an important domain for studying the basic science of neuroplasticity, as well as being implicated in many rehabilitation and occupational applications^1,26–30^. In the perceptual learning domain the overwhelming majority of research has examined discrete choice outcomes of perceptual decision-making. This is despite the fact that disregarding an important behavioral variable like RT is inefficient, and may even be misleading due to the potential for biased estimates of changes in performance^31^.

A small number of papers have reported investigations of DDM analyses of learning in perceptual decision-making tasks. Yet, results establishing the extent to which certain parameters change on different timescales are especially important but remain rare. In an early example in which monkeys completed a visual discrimination task with oculomotor responses, changes in DR were found to be related to learning-related performance improvements^32^. Human perceptual learning studies have provided similar evidence regarding training-related changes in DR^23,31^.

Other task manipulations utilizing DDM frameworks have investigated the extent to which various types of instructions affect parameter estimates over the course of learning. For example, when explicitly directing learners to emphasize either speed or accuracy in perceptual learning, the effects were best characterized by a DDM model that allowed for between-session variations in DR, RB, and NDT^20^. While overall NDT did not reliably change over time, there were main effects of learning (comparing session-level parameters) indicating decreases in RB and increases in DR. In a shorter-timescale study^33^, across 24 short blocks (approximately 1 min each), participants’ RB tended to converge on an optimal level, according to a rate-of-reward calculation. This latter result indicates that rapid measurable changes may occur in RB on timescales that are too short to be typically associated with perceptual learning, as well as too short to be measured when using standard methods of aggregating over large blocks to measure learning.

Diffusion models of learning have also been used in conjunction with other measures such as functional neuroimaging. In one such study of dot-motion direction discrimination training, specific increases in trained-stimulus DR were observed and related to fMRI activation in supplementary eye field and in ventral premotor cortex, while nonspecific increases in RB were also observed, and model comparisons did not support training-related changes in NDT^34^. In another study, cognitive training led to increases in DR and decreases in RB, although the only associations between training-related changes in DDM components and neural measures was a link between changes in RB and those in striatal activity^35,36^. The relations between DDM parameters and neural change thus clearly have the potential to be powerful tools for the cognitive neuroscience of learning, yet the relative lack of basic research on the influence of experience on DDM parameters means that tests of such correspondences remain largely under-constrained.

In all, previous investigations of perceptual learning using DDMs have shown the utility of evidence-accumulation models for understanding the mechanisms of change occurring during such learning. One potentially major limitation of these lines of research is that they have utilized analysis methods that necessarily instantiate certain implicit assumptions about the time-course of performance and learning. Specifically, assessments of changes in both DR and RB have often involved comparing parameters that were fit to full blocks or sessions of data. That is, if a study involved four separate days of learning with each day containing 700 trials, the typical analysis approach would involve fitting a separate DDM model to each participant’s and day’s data—aggregating across all 700 trials within the given days—and then using day-to-day changes in parameter estimates to make inferences about learning. This is problematic because such an approach is implicitly assuming that all trials within a day are the same, or in other words that there is no learning occurring within each day, and that learning can only manifest between days. As such, little is known about the more precise time scales at which within-day and between-day parameters changes may occur and how these changes may interact.

Aggregation-based approaches to assessments of perceptual learning, and their associated issues, are not unique to studies that have utilized DDM-type models. Instead, aggregation-based approaches have been commonplace throughout the perceptual learning literature (e.g., fitting a threshold for each individual block or session; using a point estimate from an adaptive staircase). More recent work has shown that by eschewing aggregation, and instead directly modeling perceptual ability as a function of the smallest unit of training time (i.e., trial number), core questions of theoretical interest in perceptual learning can more directly be addressed regarding the influences of experience on perception^13,37–39^. For example, different forms of generalization that result from perceptual training have been dissociated using by-trial models, with generalization in the form of learning to learn (i.e., improved learning rate) being distinguished from immediate improvements in perception^12^.

One major reason that well-calibrated models of learning are of particular importance in the study of perceptual learning is that accurately identifying the functional form that learning takes can allow for significant inferences about the underlying processes, both at behavioral and mechanistic levels^3,5,6,40^. This is particularly true in cases wherein learning takes place across multiple days. If experience-dependent change occurs during both the actual training and in between-session consolidation, then both features of the learning trajectory should be incorporated into the model of change. Alternatively, if only the overall amount of task experience influences perceptual abilities (e.g., cumulative trial number across all days), then modeling between-day discontinuities may lead to unnecessary model complexity and overfitting.

Such questions are especially relevant for learning in perceptual decision-making, an evidence accumulation process, wherein the underlying DDM parameters may change at different time scales. Indeed, although timescales of learning in perceptual training can be revealing, they are often also contentious. While certain perceptual learning experiments have involved only a few hundred trials^41^, other experiments have discarded hundreds of trials as “task learning” and instead only considered subsequent (often extensive) trajectories of change. This latter view, in which a full trajectory of performance is first dominated by “task learning” and only later by “perceptual learning,” would predict that changes in perception should be characterized by a shorter and then a longer timescale of improvement. Yet, such predictions have not been consistently borne out in analyses of the mathematical properties of perceptual learning curves^3,24^. Nonetheless, other sources of evidence have indicated that processes underlying perceptual improvements may be changing on different timescales. Neuroimaging studies have indicated, for example, that initial plasticity in sensory areas precedes larger-scale changes in connectivity^42^. A phenomenon that is perhaps even more striking is the role of sleep-associated consolidation for successful perceptual learning^43–45^. In an extreme case, if changes were to be completely reliant on sleep, then within-day performance would be fully stationary and learning curves would be dominated by between-day discontinuities. Such a proposition is incompatible with a large amount of empirical evidence^3,24,33,39^. At the opposite extreme, completely continuous trajectories of learning and a total absence of between-session discontinuities would seem equally incompatible with published findings. How, then, could the two patterns of results be resolved?

The use of evidence-accumulation models and joint distributions of RT and accuracy provide a great opportunity in this domain, as they integrate more information than either data source alone, and they do so in such a way to retain interpretability of parameters. Prior evidence for unitary processes of change^3,24^ may thus be compatible with evidence for multiple timescales of change or for between-day discontinuities in performance^43,45,46^, with different dynamics being borne out in different components of the evidence-accumulation model’s parameters. That is, both between-day discontinuity and between-day continuity may be present in processes involved during perceptual learning, but these co-existing timescales of change may only be evident when utilizing analyses of perceptual abilities that allow for process-level decompositions of performance on multiple timescales.

The current work modeled learners’ perceptual performance using a Wiener diffusion process that could take several possible forms of change over time. Data was used from a previously published^47^ computerized behavioral task that required participants to respond whether a field of dots was moving leftward or rightward as quickly and accurately as possible. On each of four separate days, participants completed 700 trials of this task (100 trials of each of 7 levels of motion coherence, see Methods; note that in the published work^47^, only the final day of training was utilized). Auditory feedback regarding accuracy was provided on each trial.

**Fig. 2:**
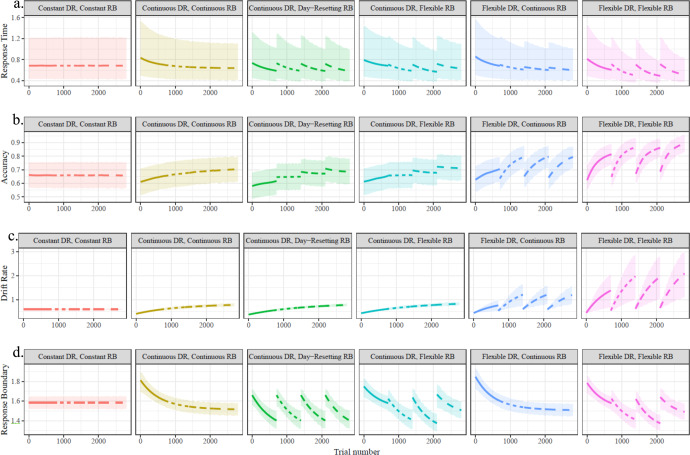
Example trajectories of exponential change in DR and RB, and their implications for observed behavior (i.e., Response Time and Accuracy). Characteristic effects can be observed (e.g., accuracy [**b**] increases with increasing DR [**c**] or increasing RB [**d**]; RT [**a**] decreases with increasing DR or decreasing RB), although the strengths of specific links may vary across the different levels of stimulus coherences. The values shown here were the fixed-effect posterior distribution estimated values and 95% CI from models reported in the Results, evaluated at the median stimulus coherence for the sole purpose of illustration (see also Supplementary Note section Best-fitting model summary output and Tables 1–5).

In line with the work reviewed above, DR and RB were of primary interest. Four candidate dynamics of change (in DR, RB, or both) were considered (see Fig. 2 for examples). The first possibility for each parameter is a fully stationary or “*constant”* process, wherein a parameter does not change across the four days of participants’ training on a 2AFC dot-motion direction discrimination task. The second possibility (“*continuous*”) involved change in the parameters as a continuous exponential function of overall experience or trial number. The third (“*day-resetting*”) allowed a within-day continuous change (as a function of within-day trial number) that repeated for each day, implying a transient and consistent divergence from baseline due to within-day task experience but a reset to that baseline between days. The fourth (“*flexible*”) allowed a within-day continuous change that also had the same speed (a time constant defining the shape of the exponential function), but independent starting and asymptotic levels each day, thereby providing a great deal of flexibility in capturing both within-day and between-day changes. Models with *constant* parameters were nested within all other models, the *day-resetting* form of change was nested within the *flexible* change, and the *continuous* change could be imitated well by the *flexible* form of change.

The different forms of change, and their combinations, implicate certain patterns of change in the processes underlying perceptual decision-making. While we expect that DR should increase with experience due to improvements in perceptual sensitivity^20,32^, our methods further allow for the adjudication of whether this change is driven by between-day disjunctions (possible in *flexible DR* models) or independent of such disjunctions as assumed in *continuous DR* models. Further, while both long-term^20^ and short-term^33^ modulations in RB have been observed, we explicitly test whether these dynamics are part of the same underlying multi-session trajectories (*continuous RB)*, driven primarily by within-day changes away from baseline levels (*day-resetting RB*), or involve both within-day and between-day changes (*flexible RB*).

When considering that DR and RB could take different forms of change, we thus ended with 5 possible dynamic models (that are plausible given previous empirical and theoretical work on learning) plus a sixth static model for comparison. These 5 models included [1] a *continuous DR, continuous RB* model with dynamics of both parameters driven by cumulative overall experience and uninfluenced by between-day discontinuities, [2] a *flexible DR, flexible RB* model with changes in both parameters varying by both within-day and between-day effects, and the corresponding [3] *continuous DR, flexible RB* and [4] *flexible DR, continuous RB* models which allow changes due to overall experience in both models but only allow between-day discontinuities or idiosyncrasies in a single DDM parameter. An additional combination of [5] *continuous DR* and *day-resetting RB* was tested to rule out the possibility that the performance of the *continuous DR, flexible RB* model may be attributable to purely resetting within-day changes in RB, with no between-day differences in RB.

We first estimated models with the above-described combinations of possible parameter changes. We then identified which model provided the best fit to our visual dot-motion discrimination perceptual learning data. Fully Bayesian nonlinear mixed-effects model estimation and comparison allowed for a robust and replicable modeling framework, given our use of publicly-available statistical packages (see Methods). Additional robustness provided by Bayesian models comes from their inherent quantification of uncertainty around each parameter estimated, particularly when the possible correlations between parameters are not known.

To preface our results, we found that the model allowing for the most flexible change in both DR and RB fit better than most others, including the model that assumed unchanging parameters across the entire timescale of the experiment. Notably however, one simplification of this most-flexible model further improved fit indices. The overall best model included increases in DR as a relatively simple and constrained exponential function of overall trial number, without between-day discontinuities, while for RB, flexible, within- and between-day variations were modeled. Compatible results were found when fitting separate models to each participant rather than all participants simultaneously. Whether comparing the full mixed-effects nonlinear models or separate by-participant nonlinear models, multiple methods of model comparison supported the notion that perceptual learning may be best understood as resulting from a continuous DR change accompanied by flexible changes in RB.

## Results

We first assessed all models’ convergences, then compared the models using change in LOOIC (approximation to leave-one-out cross-validated deviance^48^) as well as Bayes Factors (estimated using bridge sampling; see Methods). All models converged (maximum fixed-effects R-hat < 1.03), with the winning model having a minimum tail effective sample size of 311 and no divergent transitions. For fixed-effects estimates of all models, we refer the reader to Supplementary Note (section *Best-fitting model summary output* and Tables 1–5). We also assessed whether learning was evident on all stimulus coherence levels; by-participant aggregated comparisons indicated that RT reliably decreased for all coherence levels. Accuracy meanwhile increased numerically for all coherence levels, however, this was only statistically reliable for intermediate coherence levels (see also Supplementary Figs. 4 and 5).

### Model comparison results

**Fig. 3:**
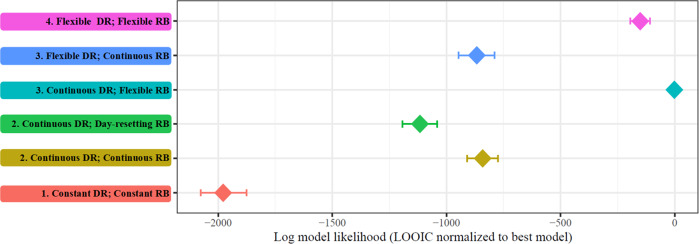
Results of model comparison. LOOIC were rescaled (inverse so to be interpreted as a relative likelihood) and normalized to the best-fitting model, which is represented by 0. This scaling provides interpretability as an approximation to a log Bayes Factor, to the extent that the LOOIC is analogous to the predictive probability provided by a Bayesian Information Criterion (BIC^55^). Alternatively, differences larger than 4 that are also larger than several standard errors (i.e., all differences in this Figure) are interpretable as indicating improvement in model fit from one model to another^57^. Numbers indicate a general ranking of model complexity. Error bars indicate the standard error of the cross-model pointwise LOOIC predictive density.

We first compared the most complex model (*flexible* DR *flexible* RB) to the simplest model (*constant* DR *constant* RB), finding that the model allowing *flexible* change in parameters over time fit better (∆_LOOIC_ = −3650.5). Subsequent model comparisons tested whether a more parsimonious form of change in which DR and RB were *continuous* functions of overall experience could effectively account for the perceptual learning observed. The *continuous* DR, *continuous* RB model did not improve model fit compared to the *flexible* DR *flexible* RB model (∆_LOOIC_ = 1383.1), nor did the *flexible* DR, *continuous* RB model (∆_LOOIC_ = 1434.1). In contrast, a model in which DR was a *continuous* function of overall trial number while RB *flexibly* changed did improve model fit over the more complex (*flexible* DR *flexible* RB) model (∆_LOOIC_ = −301.2). A different constraint on RB, that of a repeating continuous function of within-day trial number (*continuous* DR *day-resetting* RB) did not fit better than the *continuous* DR *flexible* RB model (∆_LOOIC_ = 1931.6; see Fig. 3 for a plot of all fits’ relative likelihoods).

Using the LOOIC criterion, the best model therefore included a *continuous* DR change and a *flexible* change in RB. Using Bayes Factors estimated using model-pairwise bridge sampling, very similar model comparison results were found (see Table 1). The same model (*continuous* overall-trial DR and *flexible* RB) fit better than each of the other models.

**Table 1:** Bayes Factor (base-3 log) comparisons of models using 15 runs of bridge sampling, with the most equivocal being reported.

	A	B	C	D	E	# wins
F. Flexible DR, Flexible RB	Inf	382.6	628	−119.9	468.5	4
E. Flexible DR, Continuous RB	Inf	−58.2	137.4	−591.6		2
D. Continuous DR, Flexible RB	Inf	547.6	Inf			5
C. Continuous DR, Day-resetting RB	628	−210.7				1
B. Continuous DR, Continuous RB	Inf					3
A. Constant DR, Constant RB						0

Positive numbers indicate that the row model fit better, while negative numbers indicate that the column model fit better. Inf indicates that the bridge sampling procedure could not effectively estimate the relative evidence between models, with the row model being preferred. The last column shows the number of pairwise comparisons in which the row model was preferred (5 being the best, 0 being the worst). These rankings align with the rank order of the models in Fig. 2, notably, with the same best-fitting model with continuous DR and flexible RB.

### Characteristics of the best-fitting model

The best-fitting model recovered well both accuracy (*r* = 0.76) and response time (*r* = 0.56; see Methods and Supplementary Figs. 2 and 3). This model indicated an improvement (increase) in DR as an exponential function of overall trial number throughout the whole experiment (i.e., trials 1 through 2800). Model fixed effects indicated that DR increased with training (start mean = 0.44, se = 0.05; asymptote mean = 0.96, se = 0.11), with a sample-level time-to-half-of-learning of 1261 trials. Every participant’s DR was estimated as increasing (of participants’ point estimates, change mean = 0.516, sd = 0.175, min = 0.209, max = 0.800).

**Fig. 4:**
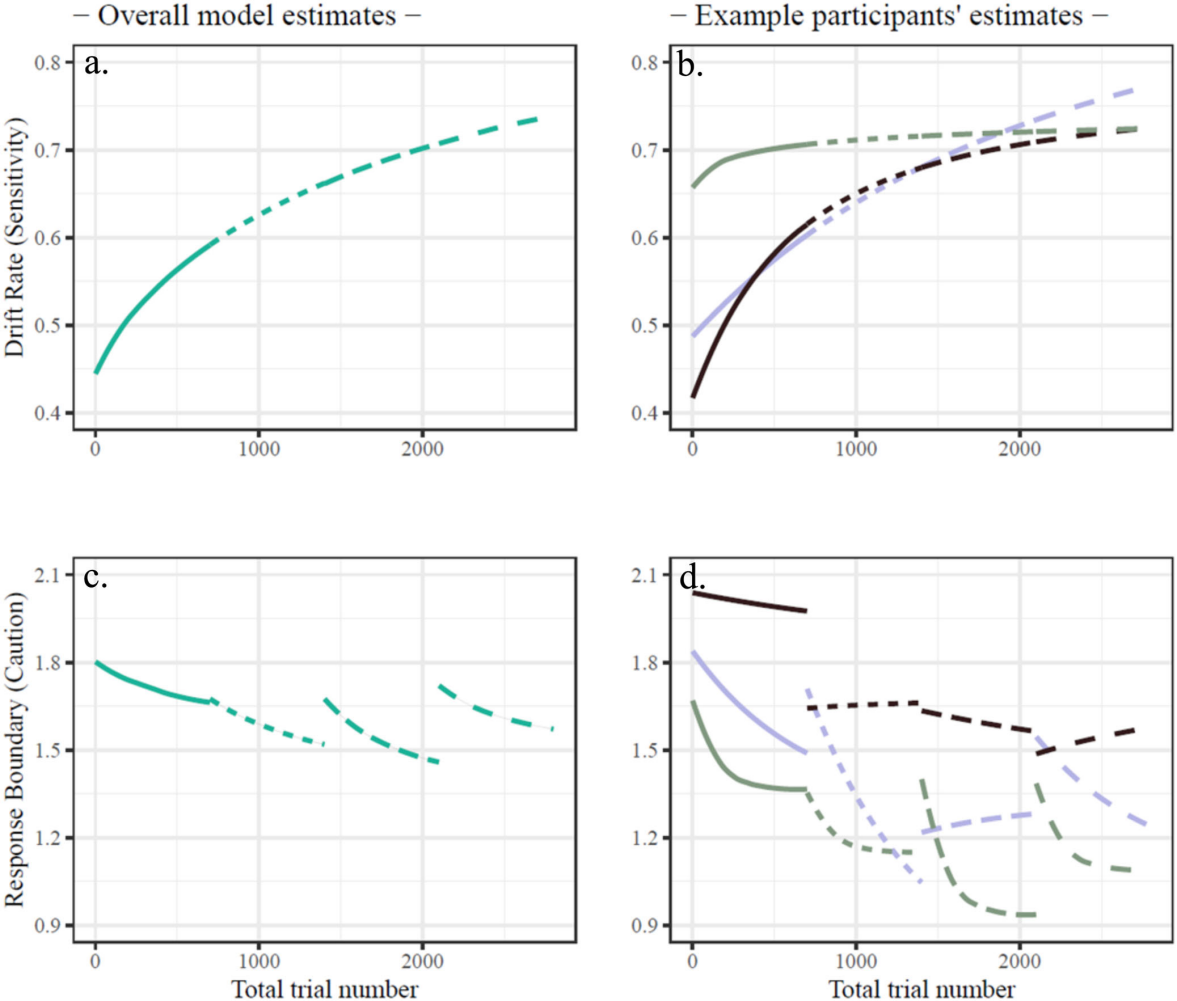
Fitted point estimate values of the best-fitting model. For both DR [**a**, **b**] and RB [**c**, **d**], left panels [**a**, **c**] show the overall fits to all participants (see also Fig. 1). The right panels [**b**, **d**] show fits for three example participants (chosen to illustrate patterns of change and heterogeneity). Each day is represented by a different line type.

Response boundary parameters changed less systematically across days and participants (see Fig. 4). While at the group level slight decreases appeared to occur, with some “resetting” between days, interpreting such group-level effects is difficult due to the large amount of inter- and intra-individual variability^49^. Within-day decreases in RB were present in most participants (80.9%, 61.9%, 76.2%, and 55.1% of participants, on each of the four days respectively). Between-day changes in RB were primarily decreasing from day 1 to day 2 (95.2% of participants), but only a minority of participants decreased from the end of day 2 to the beginning of day 3 or the end of day 3 to the beginning of day 4 (28.6% of participants in each transition).

### By-participant model fits

We fit the same set of models, without the mixed-effects structure, to each participant’s data separately and compared the within-participant results in the same manner. The *continuous DR flexible RB* parameterization was the best-fitting model in 19/21 participants using LOOIC comparisons and 15/21 participants using Bayes Factor comparisons. There was also a fairly high correspondence between the by-participant point estimates of parameters, when comparing models fit to participants separately and the best-fitting model fit to all participants simultaneously (e.g., time constant of change in DR Pearson *r* = 0.77, asymptotic DR *r* = 0.85). Each of these comparisons thus reinforces the results of the full mixed-effects models. See Supplementary Note for further details.

### Logistic model fits

In order to provide a comparison to more typical approaches to perceptual learning research (i.e., that typically only consider participant accuracy), we fit several models using continuous-time changes in thresholds of logistic psychometric functions^39,50^. These models were fit to only accuracy data rather than the joint RT and accuracy distributions. Candidate models of change in by-trial threshold included the *continuous, day-resetting* and *flexible* forms described in the primary analyses, as well as an additional combination of *continuous* and *day-resetting* forms (i.e., adding a day-resetting trajectory offset to the continuous function). The best-fitting model was this last one, with an intermediate level of complexity (LOOIC = 61182.7). The other models, in decreasing order of goodness-of-fit, were *flexible* (LOOIC = 61202.6), *continuous* (LOOIC = 61238.6), and *day-resetting* (LOOIC = 61352.3). These results, as in the main results, supported an intermediate-complexity model involving two timescales of change. Yet, unlike the DDM models, the logistic fits were unable to provide any mechanistic account regarding what changed on which timescale. While the lack of consistent improvements in accuracy on the smallest and largest coherence levels means that these results are largely driven by accuracy changes at intermediate coherence levels for most participants (see Supplementary Fig. 4), this limitation of psychometric function fits further demonstrates the utility of understanding the joint distributions of RT and accuracy during learning (i.e., due to the improvements in RT on all coherence levels; see Supplementary Fig. 5). See Supplementary Note for further details.

## Discussion

Here we used nonlinear mixed-effects DDMs to characterize within-day and between-day changes during a perceptual decision-making task. Changes in DR were found to be best modeled as an exponential function of the number of trials over 4 different days of training. In contrast, changes in RB were best modeled by heterogeneous dynamics with both continuous within-day changes and unsystematic between-day variations in trajectories of change. Such findings support the conclusions of earlier work identifying DR as an index of perceptual learning in monkeys^32^ and humans^31,34^ while adding additional specificities in adjudicating between timescales of change.

More broadly, this work corroborates the rapid adjustments of RB observed in perceptual decision-making^33^ while providing a possible unifying explanation for two seemingly contradictory observations in perceptual learning: Improvements as a continuous function of experience^39^ and large between-day discontinuities^43^. The present work suggests that each of these phenomena may be occurring at their own time scales. Importantly, it points to the different processes mediating decision making (DR and RB in particular) changing with rather different dynamics. Previous work has identified crucial roles for inhibitory neural activity between training sessions (particularly during sleep) for increasing improvements’ resistance to retroactive interference^51^, with our results providing complementary evidence for multiple processes. While improvements in perceptual sensitivity accumulate gradually with training (e.g., associated with higher DR and with excitatory and plastic neural states), much observed behavioral variation may be in fact the result of modulations in inhibitory processes (e.g., associated with higher decision criteria RB and with inhibitory and stable neural states). That is, the speed at which inhibitory processes can be upregulated or downregulated may be much faster than the speed at which perceptual processes are modifiable, leading to much behavioral variation as well as important processes of consolidation and resistance to interference.

As such, using only impoverished discrete-choice behavioral data could prevent or bias possible inferences regarding learning trajectories^31^. For instance, robust improvements in performance occurred in accuracy for only some coherence levels whereas RT showed more widespread improvements. Integrating both of these into a single model-based approach allowed us to better parcel out sources of variation and identify changes in perceptual sensitivity and decision processes. Practically, the present work provides a clear justification for the use of diffusion models’ DR parameter as an index of perceptual learning as a function of each and every training trial. The interpretability of RB is more complex due to the presence of both within-day and between-day dynamics. While such changes are in line with RB reflecting adaptive choices during decision making, such as being more or less risk-averse, the source of such large across-day and across-participant heterogeneity remains poorly understood. Convergent measures (e.g., neuroimaging) or formalized predictions of performance (e.g., “optimal” caution levels^33^) may provide future clarity for the processes of change occurring on this faster and more disjoint timescale.

An important component of the present work is that it uses advanced yet relatively accessible quantitative methods (see Supplementary Note for the best-fitting model’s code). The increasingly widespread availability of the DDM estimation methods in statistical packages assists in providing a larger number of researchers with the tools needed to conduct the analyses reported here. While the computational resources needed were extensive (at least several weeks of computer time for each model), that limitation should become less problematic as increasingly highly-powered computers become more commonplace. In addition, once the groundwork has been established regarding appropriate model parameterizations for learning, those models can be used as test cases to develop and assess more efficient fitting methods. We recognize that the models reported here only represent a small portion of the possible models of change in perceptual learning. Yet, we believe they are sufficient for novel and informative inferences, and we have not fit various intermediate models due to their likely lack of informativeness and the extensive computational resources necessary to estimate model parameters.

As with the wide applicability of DDMs to cognitive as well as perceptual processes, the approach applied here has applications far beyond learning in visual motion perception. Using DDMs to better understand mechanisms contributing to trajectories of learning may be useful in word learning^52^, cognitive training, or other learning contexts in which information must be quickly and accurately acted upon.

Perceptual learning holds many translational promises as well as acting as a window into mechanisms of adult neuroplasticity, yet understanding perceptual learning is predicated on appropriate inferences from learners’ perceptual decision-making. By using a formal evidence-accumulation model of perceptual decision-making, seemingly contradictory propositions regarding trajectories of learning can be explained: Different components of evidence accumulation may be changing within training sessions and between days on independent timescales. Here we have presented evidence that perceptual sensitivity (DR) changes as a continuous function of trial number, while the amount of evidence needed to elicit a decision (RB) dynamically changes both continuously within a training session and unsystematically between days.

## Methods

### Participants and procedures

Data were archival and only secondary data analyses were conducted for this paper; procedures were originally approved by the University of Rochester ethics board and informed consent was obtained from each participant. Data, originally reported in Green, Pouget, and Bavelier^47^, were collected from 21 male young adult participants completing 4 consecutive days each of a dot-motion discrimination task^53,54^. Only the last day’s data were reported in the original paper. Participants were originally included to fall into either an “action video game player” group (mean age = 18.8) or a “not action video game player” group (mean age = 20.6). One participant was excluded from the original sample of 22 due to an unexplained very long response time distribution on one session. The current paper is not primarily concerned with possible differences between game player groups, which will not be addressed, although every model does include between-subject fixed effects controlling for potential differences between groups on each diffusion model parameter (see below). Such fixed effect parameters should mitigate possible bimodality in participant-level parameters. Stimuli were presented on a 75 Hz CRT monitor using Psychtoolbox (Brainard 1997). Left or right coherence percentages were 0.8%, 1.6%, 3.2%, 6.4%, 12.8%, 25.6%, or 51.2%, in a randomized order.

### Modeling framework; brms multilevel nonlinear fits with various timescales

To fit by-trial continuous-time drift diffusion models we used the **brms** package in **R**, which itself uses the **Stan** Bayesian modeling framework^17–19^. Models were, in essence, nonlinear generalized regressions of the joint RT and accuracy distributions, with each trial’s drift diffusion parameters being either static over all trials or a function of trial number (parameterized in several different ways; see the next section). In turn each model’s DDM parameters, whether unchanging or related to specific components of time-related change, were themselves the fit value of a generalized linear mixed-effects model (estimated in parallel see ref. ^38^). Such a framework allowed for simultaneous estimation of all parameters for all participants. Another crucial benefit of our approach was that, because **brms** includes default priors for Wiener diffusion generalized linear models, the only priors necessary to manually specify were those related to the nonlinear components of models (i.e., amount of time taken to change, and an NDT with a small additive offset of 0.001 seconds). Default priors improve the direct applicability of our implementation to novel datasets and increase the ease with which others may conduct such analyses, while coming at the cost of a likely decrease in efficiency and greater difficulty interpreting our Bayes Factor results (i.e., because priors were not explicitly adapted to our dataset or hypotheses). For details see the Supplementary Note.

Regardless of the exact combination of parameter changes implemented, changes were always defined as a DDM parameter being an exponential function of trial number (see Eq. 1). Every participant had the model’s set of DDM parameters estimated for them, utilizing the mixed-effects structure described above (i.e., by-participant random intercepts). In this parameterization the time constant of change (*rate*, or inverse speed) was independent of both the *start* and *asymptote* of a given parameter. The critical differences between models being compared involved the timescale of change (i.e., whether *trialNumber* was within-day trial number or cumulative across all days) and, if within-day trial number was considered, whether *start* and *asymptote* parameters were shared across all days or whether they were allowed to vary across days. The *rate* parameter was the binary log of a time–to-50%-of-change constant. The constant 2 added to the rate prevented trajectories from indicating 50% of change in less than 2 trials, which assists in model identifiability and estimation. Note that *param* may be DR or RB.

1 $${param}={asymptot}{e}_{{param}}+({star}{t}_{{param}}-{asymptot}{e}_{{param}})* {2}^{(1-{trialNumber})/(2+{2}^{{rat}{e}_{{param}}})}$$

### Models being compared

Models being compared began with “constant” models in which parameters did not vary by time. In this comparison, coherence was either linear (untransformed) or log-transformed, then median-centered. Our comparison between these two models established the empirical basis for the linear relationship between DR and coherence^53^. In this comparison to determine the scaling of stimulus strength with relation to DR, we found that the constant-parameter model with a log-transformed stimulus coherence DR fit much worse than the same model with DR as a linear function of coherence (∆_LOOIC_ = 3781.3). For this reason, all subsequent analyses used a linear function of coherence.

Parameters were then allowed to vary as a continuous function of overall trial number (*continuous*) or continuous function of within-day trial number with within-participant across-day constant starts and asymptotes (*day-resetting*). The most complex model (*flexible*) allowed for a within-day change with separate starts and asymptotes for each day; each participant only had one rate, to assist in estimation.

These forms of change allowed for model comparisons to adjudicate between distinct potential mechanisms for experience-dependent change. A mechanism of change due solely to cumulative experience with the task, or law of effect, would benefit the *continuous* form of change over the *flexible* form of change due to its greater parsimony (i.e., smaller parameter number). Similarly, a mechanism of change involving a within-day perturbation from each participant’s baseline and a between-day return to that baseline would be indicated by the *day-resetting* parameterization of change, which is also more parsimonious than the fully *flexible* form of change. In contrast, if model comparisons support *flexible* trajectories for a parameter, it indicates that the mechanisms of change are likely to be heterogeneous for that parameter. Many possible generative models are compatible with this *flexible* form of change, and selectively applying constraints (as the *continuous* and *day-resetting* models do) provide tests of whether the complexity of the *flexible* model is warranted or if the more parsimonious and mechanistically interpretable models are justified.

As described in Eq. 1, parameters were defined as 3-parameter exponential functions of time with starting values, asymptotic values, and a 50%-of-change time constant defining the rate of change^3,24,37^. *Continuous* change and *day-resetting* change each estimated one *start*, *rate* and *asymptote* for each participant; the two forms of change only differed on the timescale of that change. *Flexible* change estimated one *rate* for each participant across the four days, plus one *start* and one *asymptote* for each day for each participant. All priors were default where possible, with the *rate* parameters having normal priors centered on 25% of the maximum trial number (i.e., 175 for within-day change and 700 for full-experiment change). These priors’ SD was 1, thereby leading most of the density (i.e., between +2 and -2 SD) to be between the full timescale of change and 1/16th of that timescale of change.

### Model specification and priors

We report model formulas in the common “Wilkensen” format, for clarity and similarity to the **R** implementation (example: driftRate ~ predictorA + (predictorB | groupingVariable)). In this syntax the predicted variable (here, the drift diffusion parameter) is to the left of a tilde. The predictors are to the right of the tilde. A 1 indicates an estimated intercept value. Components of the predictors in parentheses are random effects, with predictors to the left of the vertical bar and the grouping variable to the right of the vertical bar.

All models had the following shared features:

Both action video game players (AVGP) and non-action video game players (NVGP) were included, with fixed main effects controlling for any inter-group differences on each parameter in any given model. (All parameters also have participant-level estimated coefficients). Trials with RTs below 0.16 or above 2.5 were excluded. Time-evolving components were estimated as binary log (i.e., base-2) of the time constant to 50% of change.

Drift rate was estimated with no link function. The prior was set to the default **brms** prior for the drift rate (given our data), student_t(3,1,10). Across models, drift rate was always estimated with a fixed-effect intercept, a by-subject intercept, and a by-subject slope for median-centered coherence (i.e., drift rate varied linearly, by subject, as a function of stimulus coherence). Models were tested that included either “raw” coherence percentages or their log transforms, in order to test the assumption that the relation between coherence and drift rate would be linear. Example formulas: fixed drift rate: drift_rate ~ 1 + VGPstatus + (coherence || subj), asymptote of a time-evolving drift rate: drAsym ~ 1 + VGPstatus + (coherence || subj).

Response boundary was estimated on a log scale. The prior for all models was set to the default **brms** prior for the response boundary (given our data), normal(-0.6, 1.3). By-subject intercepts and fixed-effects intercepts were estimated. Example formulas: fixed response boundary: response_boundary ~ 1 + VGPstatus + (1 || subj), asymptote of a time-evolving response boundary: rbAsym ~ 1 + VGPstatus + (1 || subj).

Non-decision time was estimated using an exponential distribution with an offset of .001 and a mean of .15. This offset approach constrained sampling to plausible values and improved model efficiency. To the extent that all models use this approach, there should not be any bias introduced into any models or comparisons. Fixed-effect intercepts as well as by-subject intercepts were estimated. Example formula: ndt ~ 0.001 + ndtOffset; ndtOffset ~ 1 + (1 || subj). The bias term was estimated with a fixed-effect intercept and a by-subject intercept (i.e., bias ~ 1 + VGPstatus + (1 || subj)). The bias was estimated on a logit scale, and its prior was normal(0,1).

### Model assessment

Given the above forms of change, we tested combinations of time-varying DR and RB parameters (see Introduction). While we considered testing time-varying non-decision times as well, we decided not to include these models in our current manuscript due to the likely possibility that empirical changes in non-decision time parameters may reflect spurious patterns due to a lack of recoverability between non-decision time and RB^14^. Primary model comparison used an efficient approximation to leave-one-out cross-validation^48^ (see also the documentation for the **loo** R package). Using this method we report the LOO information criterion (LOOIC), which is on a deviance scale (i.e., lower values are better) and is interpretable similarly to an AIC or BIC value see, e.g.^55,56^^,^. Further, models’ LOOIC difference values larger than 4 are interpretable in terms of the number of standard errors of the differences^57^.

Additional model comparison used Bayes Factors estimated using bridge sampling^58^. Fifteen bridge sampling runs were completed using a warped multivariate normal proposal distribution, which is more robust than the standard multivariate normal proposal distribution. Bayes factors were transformed with a base-3 logarithm so that conventional model selections thresholds (i.e., 3 times as much evidence for one model over the other being “substantial evidence”) would coincide with cutoffs at -1 and +1. Of the 15 bridge sampling runs, we report the most equivocal (i.e., BF_log3_ closest to 0).

Absolute model fit (i.e., recovery of raw data) was assessed by binning the data into 25-trial blocks (112 blocks total per participant) and averaging RT and accuracy within each block for each coherence level. Both the data and predicted values from the best-fitting model followed this procedure. Given these binned averages, zero-order product-moment correlations between the data and the model predictions served to indicate model recovery of patterns in the raw data. Further visualizations can be seen in Supplementary Figs. 2 and 3.

### By-participant model fits

Additional comparisons fit each of the 6 models compared in the main Results to participants separately, and used within-participant Bayes Factors and LOOIC. By-participant fits used the same set of models (formulas and priors, as relevant to single participants) as the mixed-effects models fit to all participants simultaneously. Likewise, comparisons used LOOIC comparisons and Bayes Factors using bridge sampling. All indices indicated convergence.

### Logistic model fits

Comparisons to more conventional methods of fitting perceptual decision-making data (i.e., considering only accuracy) were implemented using logistic psychometric functions^50^. Logistic models utilized an approach that conforms more closely to classical analyses of psychophysics, that is, fitting a logistic psychometric function linking coherence to the probability of a participant responding that motion was in a certain direction. A lapse rate of 1% was used. Like in the main results, performance (here, threshold) was modeled as a continuous exponential function of time, with “time” corresponding to within-session trial number, overall trial number, or a combination of the two. Response times were z-scored and by-participant random slopes of response times were estimated for each of the starting and asymptotic threshold parameters, as a rough method of including all of the information in the logistic models that had also been included in the DDM models

### Reporting summary

Further information on research design is available in the Nature Research Reporting Summary linked to this article.

## Supplementary information

Supplementary Note

Reporting Summary

## Data Availability

All data are available at 10.5281/zenodo.7025263.

## Code availability

Model code is available upon request to the corresponding author.

## References

[1] Deveau J, Lovcik G, Seitz A (2013). The therapeutic benefits of perceptual learning. Curr. Trends Neurol..

[2] Mayer RE (2001). What Good is Educational Psychology? The Case of Cognition and Instruction. Educ. Psychol..

[3] Dosher BA, Lu Z-L (2007). The functional form of performance improvements in perceptual learning: learning rates and transfer. Psychol. Sci..

[4] Gold JI, Ding L (2013). How mechanisms of perceptual decision-making affect the psychometric function. Prog. Neurobiol..

[5] Heathcote A, Brown S, Mewhort DJ (2000). The power law repealed: the case for an exponential law of practice. Psychon. Bull. Rev..

[6] Newell, A. & Rosenbloom, P. S. Mechanisms of skill acquisition and the law of practice. in Cognitive skills and their acquisition (ed. Anderson, J. R.) 1–51 (Lawrence Erlbaum, 1981).

[7] Heathcote A, Hayes B (2012). Diffusion versus linear ballistic accumulation: Different models for response time with different conclusions about psychological mechanisms?. Can. J. Exp. Psychol./Rev. canadienne de. psychologie exp.érimentale.

[8] Pedersen ML, Frank MJ, Biele G (2017). The drift diffusion model as the choice rule in reinforcement learning. Psychon. Bull. Rev..

[9] Ratcliff R (1978). A theory of memory retrieval. Psychol. Rev..

[10] Ratcliff R, Tuerlinckx F (2002). Estimating parameters of the diffusion model: approaches to dealing with contaminant reaction times and parameter variability. Psychon. Bull. Rev..

[11] Gallistel, C. R. The Organization of learning. (MIT Press, 1993).

[12] Kattner F, Cochrane A, Cox CR, Gorman TE, Green CS (2017). PerceptuaL Learning Generalization from Sequential Perceptual Training as A Change in Learning Rate. Curr. Biol..

[13] Zhang P, Zhao Y, Dosher BA, Lu Z-L (2019). Assessing the detailed time course of perceptual sensitivity change in perceptual learning. J. Vis..

[14] Dutilh G (2019). The quality of response time data inference: a blinded, collaborative assessment of the validity of cognitive models. Psychon. Bull. Rev..

[15] Frank MJ (2015). fMRI and EEG Predictors of Dynamic Decision Parameters during Human Reinforcement Learning. J. Neurosci..

[16] Vandekerckhove J, Tuerlinckx F, Lee MD (2011). Hierarchical diffusion models for two-choice response times. Psychological Methods.

[17] Bürkner P-C (2017). **brms**: An *R* Package for Bayesian Multilevel Models Using *Stan*. J. Stat. Soft.

[18] Stan Development Team. Stan Modeling Language Users Guide and Reference Manual. (2022).

[19] Stan Development Team. Rstan: the R interface to Stan. (2022).

[20] Zhang J, Rowe JB (2014). Dissociable mechanisms of speed-accuracy tradeoff during visual perceptual learning are revealed by a hierarchical drift-diffusion model. Front. Neurosci..

[21] Shadlen MN, Kiani R (2013). Decision making as a window on cognition. Neuron.

[22] Mulder MJ, Wagenmakers E-J, Ratcliff R, Boekel W, Forstmann BU (2012). Bias in the brain: a diffusion model analysis of prior probability and potential payoff. J. Neurosci..

[23] Petrov AA, Van Horn NM, Ratcliff R (2011). Dissociable perceptual-learning mechanisms revealed by diffusion-model analysis. Psychon. Bull. Rev..

[24] Cochrane A, Green CS (2021). Assessing the functions underlying learning using by-trial and by-participant models: Evidence from two visual perceptual learning paradigms. J. Vis..

[25] Fahle M (2005). Perceptual learning: specificity versus generalization. Curr. Opin. Neurobiol..

[26] Ahissar M, Nahum M, Nelken I, Hochstein S (2009). Reverse hierarchies and sensory learning. Philos. Trans. R. Soc. B: Biol. Sci..

[27] Johnston IA (2020). Perceptual learning of appendicitis diagnosis in radiological images. J. Vis..

[28] Kellman PJ, Garrigan P (2009). Perceptual learning and human expertise. Phys. Life Rev..

[29] Lu Z-L, Lin Z, Dosher BA (2016). Translating perceptual learning from the laboratory to applications. Trends Cogn. Sci..

[30] Polat U, Ma-Naim T, Belkin M, Sagi D (2004). Improving vision in adult amblyopia by perceptual learning. Proc. Natl Acad. Sci. USA.

[31] Liu CC, Watanabe T (2012). Accounting for speed–accuracy tradeoff in perceptual learning. Vis. Res..

[32] Eckhoff P, Holmes P, Law C, Connolly PM, Gold JI (2008). On diffusion processes with variable drift rates as models for decision making during learning. N. J. Phys..

[33] Evans NJ, Brown SD (2017). People adopt optimal policies in simple decision-making, after practice and guidance. Psychon. Bull. Rev..

[34] Jia K (2018). Visual perceptual learning modulates decision network in the human brain: the evidence from psychophysics, modeling, and functional magnetic resonance imaging. J. Vis..

[35] Schmiedek L, Lindenberger (2010). Hundred days of cognitive training enhance broad cognitive abilities in adulthood: findings from the COGITO study. Front. Aging Neurosci..

[36] Kühn S (2011). Brain areas consistently linked to individual differences in perceptual decision-making in younger as well as older adults before and after training. J. Cogn. Neurosci..

[37] Cochrane A (2020). TEfits: nonlinear regression for time-evolving indices. J. Open Source Softw..

[38] Cochrane A, Green CS (2021). Trajectories of performance change indicate multiple dissociable links between working memory and fluid intelligence. npj Sci. Learn..

[39] Kattner F, Cochrane A, Green CS (2017). Trial-dependent psychometric functions accounting for perceptual learning in 2-AFC discrimination tasks. J. Vis..

[40] Newell KM, Mayer-Kress G, Hong SL, Liu Y-T (2009). Adaptation and learning: characteristic time scales of performance dynamics. Hum. Mov. Sci..

[41] Poggio T, Fahle M, Edelman S (1992). Fast perceptual learning in visual hyperacuity. Science.

[42] Kang D-W (2018). Structural and functional connectivity changes beyond visual cortex in a later phase of visual perceptual learning. Sci. Rep..

[43] Karni A, Sagi D (1993). The time course of learning a visual skill. Nature.

[44] Mednick SC, Cai DJ, Kanady J, Drummond SPA (2008). Comparing the benefits of caffeine, naps and placebo on verbal, motor and perceptual memory. Behav. Brain Res..

[45] Tamaki M (2020). Reward does not facilitate visual perceptual learning until sleep occurs. Proc. Natl Acad. Sci. USA.

[46] Yotsumoto Y, Watanabe T, Sasaki Y (2008). Different dynamics of performance and brain activation in the time course of perceptual learning. Neuron.

[47] Green CS, Pouget A, Bavelier D (2010). Improved probabilistic inference as a general learning mechanism with action video games. Curr. Biol..

[48] Vehtari A, Gelman A, Gabry J (2017). Practical Bayesian model evaluation using leave-one-out cross-validation and WAIC. Stat. Comput.

[49] Brown S, Heathcote A (2003). Averaging learning curves across and within participants. Behav. Res Methods Instrum. Comput.

[50] Dale G, Cochrane A, Green CS (2021). Individual difference predictors of learning and generalization in perceptual learning. Atten. Percept. Psychophys..

[51] Tamaki M (2020). Complementary contributions of non-REM and REM sleep to visual learning. Nat. Neurosci..

[52] Dutilh G, Vandekerckhove J, Tuerlinckx F, Wagenmakers E-J (2009). A diffusion model decomposition of the practice effect. Psychon. Bull. Rev..

[53] Palmer J, Huk AC, Shadlen MN (2005). The effect of stimulus strength on the speed and accuracy of a perceptual decision. J. Vis..

[54] Watamaniuk SNJ, Sekuler R (1992). Temporal and spatial integration in dynamic random-dot stimuli. Vis. Res..

[55] Wagenmakers E-J (2007). A practical solution to the pervasive problems ofp values. Psychon. Bull. Rev..

[56] Wagenmakers E-J, Farrell S (2004). AIC model selection using Akaike weights. Psychon. Bull. Rev..

[57] Sivula, T., Magnusson, M., Matamoros, A. A. & Vehtari, A. Uncertainty in Bayesian Leave-One-Out Cross-Validation Based Model Comparison. Preprint at http://arxiv.org/abs/2008.10296 (2022).

[58] Gronau QF, Singmann H, Wagenmakers E-J (2020). **bridgesampling**: An R Package for Estimating Normalizing Constants. J. Stat. Soft..

