# Supplementary material for: Multiple timescales of learning indicated by changes in evidence-accumulation processes during perceptual decision-making
Source: NPJ Sci Learn. 2023 Jun 8;8:19. doi: 10.1038/s41539-023-00168-9 (PMC10250420; doi:10.1038/s41539-023-00168-9)
Supplement: Supplementary file 1 — Supplementary Note [file 41539_2023_168_MOESM1_ESM.docx]

# Supplementary Note

## Best-fitting model code

rt | dec(correct) ~ (drAsym + (drStart - drAsym) * (2^((1 - trial)/(2 + 2^drRate))))

bs ~ exp(rbAsym) + (exp(rbStart) - exp(rbAsym)) * (2^((1 - dayTrials)/(2 + 2^rbRate)))

ndt ~ 0.001 + ndtOffset

drAsym ~ isVGP + (coherence || subID)

drStart ~ isVGP + (coherence || subID)

drRate ~ isVGP + (1 || subID)

bias ~ isVGP + (1 || subID)

rbAsym ~ isVGP + day + (day || subID)

rbRate ~ isVGP + (1 || subID)

rbStart ~ isVGP + day + (day || subID)

ndtOffset ~ isVGP + (1 || subID)

All parameters to the left of tildes are estimated using the predictors to the right of the tildes. In the top block, the first three formulas predict the DR, RB, and NDT, respectively; these three are direct equations. The second block contains symbolic formulas in Wilkinsen notation predicting each of the parameters defined in the top block. The Wiener response distribution in **brms**/**rstan** was used, with model specifications being described in the Methods. The ndtOffset parameter had a prior defined by an exponential distribution with a mean of .15 seconds. All start and asymptote priors were defined identically to the **brms** defaults (taken from the constant model) which are by design minimally informative.

## Best-fitting model summary output

Family: wiener

Links: mu = identity; bs = identity; ndt = identity; bias = logit

Formula: rt | dec(correct) ~ (drAsym + (drStart - drAsym) * (2^((1 - trial)/(2 + 2^drRate))))

drAsym ~ isVGP + (coherence || subID)

drStart ~ isVGP + (coherence || subID)

drRate ~ isVGP + (1 || subID)

bias ~ isVGP + (1 || subID)

bsAsym ~ isVGP + day + (day || subID)

bsRate ~ isVGP + (1 || subID)

bsStart ~ isVGP + day + (day || subID)

ndtOffset ~ isVGP + (1 || subID)

bs ~ exp(bsAsym) + (exp(bsStart) - exp(bsAsym)) * (2^((1 - dayTrials)/(2 + 2^bsRate)))

ndt ~ 0.001 + ndtOffset

Data: dat_all (Number of observations: 58438)

Draws: 2 chains, each with iter = 15000; warmup = 9000; thin = 3;

total post-warmup draws = 4000

Group-Level Effects:

~subID (Number of levels: 21)

Estimate Est.Error l-95% CI u-95% CI Rhat

sd(bias_Intercept) 0.09 0.02 0.06 0.13 1.00

sd(drAsym_Intercept) 0.31 0.10 0.14 0.55 1.00

sd(drAsym_coherence) 9.27 1.68 6.57 12.92 1.01

sd(drStart_Intercept) 0.18 0.04 0.11 0.27 1.00

sd(drStart_coherence) 4.79 0.74 3.60 6.51 1.01

sd(drRate_Intercept) 2.18 0.75 0.80 3.72 1.02

sd(bsAsym_Intercept) 0.24 0.05 0.16 0.37 1.00

sd(bsAsym_dayday2) 0.35 0.08 0.22 0.54 1.00

sd(bsAsym_dayday3) 0.24 0.07 0.14 0.41 1.01

sd(bsAsym_dayday4) 0.22 0.06 0.14 0.36 1.00

sd(bsRate_Intercept) 1.46 0.34 0.92 2.23 1.00

sd(bsStart_Intercept) 0.28 0.05 0.20 0.42 1.00

sd(bsStart_dayday2) 0.15 0.03 0.10 0.22 1.00

sd(bsStart_dayday3) 0.19 0.04 0.14 0.27 1.00

sd(bsStart_dayday4) 0.19 0.04 0.13 0.28 1.00

sd(ndtOffset_Intercept) 0.06 0.01 0.05 0.09 1.00

Population-Level Effects:

Estimate Est.Error l-95% CI u-95% CI Rhat

bias_Intercept -0.30 0.02 -0.34 -0.26 1.00

drAsym_Intercept 0.96 0.11 0.76 1.19 1.00

drAsym_isVGP -0.02 0.22 -0.46 0.39 1.01

drStart_Intercept 0.44 0.05 0.34 0.53 1.00

drStart_isVGP 0.08 0.09 -0.10 0.27 1.00

drRate_Intercept 10.30 0.60 9.03 11.31 1.01

drRate_isVGP -0.98 1.01 -2.94 1.02 1.01

bsAsym_Intercept 0.41 0.07 0.27 0.53 1.00

bsAsym_isVGP 0.15 0.13 -0.09 0.41 1.00

bsAsym_dayday2 -0.14 0.09 -0.33 0.02 1.01

bsAsym_dayday3 -0.18 0.07 -0.32 -0.05 1.01

bsAsym_dayday4 -0.05 0.06 -0.16 0.08 1.00

bsRate_Intercept 8.56 0.39 7.79 9.27 1.01

bsRate_isVGP -0.11 0.63 -1.30 1.16 1.01

bsStart_Intercept 0.56 0.06 0.43 0.68 1.00

bsStart_isVGP -0.17 0.12 -0.43 0.07 1.00

bsStart_dayday2 -0.08 0.04 -0.15 -0.01 1.00

bsStart_dayday3 -0.07 0.04 -0.15 0.02 1.00

bsStart_dayday4 -0.05 0.05 -0.14 0.04 1.00

ndtOffset_Intercept 0.22 0.01 0.20 0.25 1.01

ndtOffset_isVGP -0.04 0.02 -0.09 0.00 1.02

bias_isVGP -0.12 0.04 -0.20 -0.03 1.00

Note that Effective Sample Size (ESS) estimates were removed from this output to simplify the presentation. Response Boundary is labeled bs (boundary separation) here.

## Supplementary figures

*
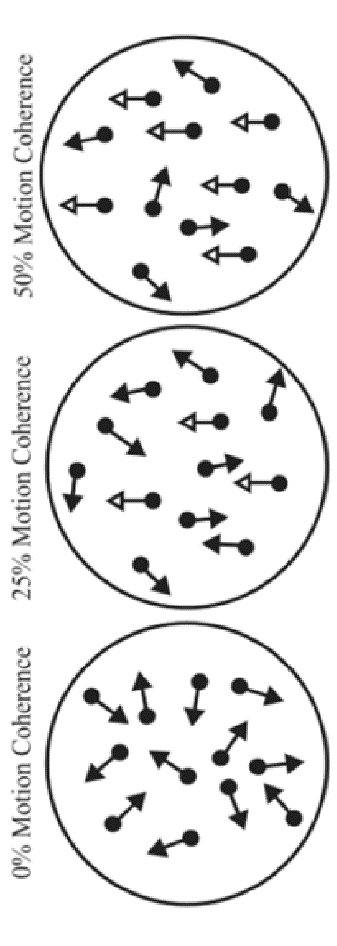
*

*Supplementary Figure 1.* ***Task schematic.*** *The behavioral task consisted of a field of stochastically moving dots within a circular aperture, with some proportion of the dots moving coherently either right or left. A large range of coherences are presented here for demonstration purposes. Figure adapted from Green, Pouget, & Bavelier (2010).*


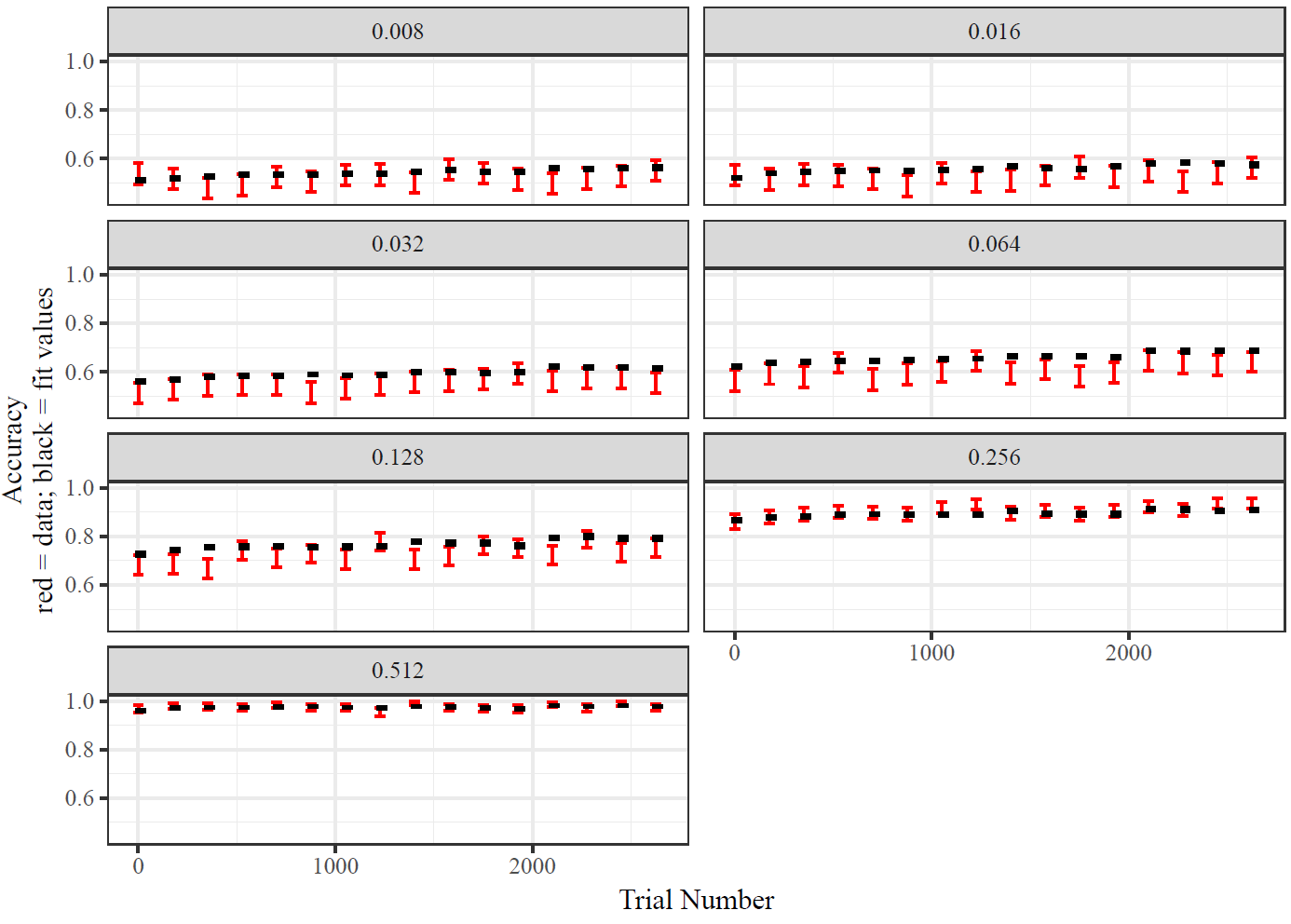


*Supplementary Figure 2.* ***Recovery of patterns of accuracy over time****. Separated by stimulus coherence level (from .008 to .512). Raw accuracies and predicted values were each binned into blocks of 175 trials with 4 blocks per day for a total of 2800 trials over 7 coherences. Error bars indicate 95% confidence intervals.*

##### *
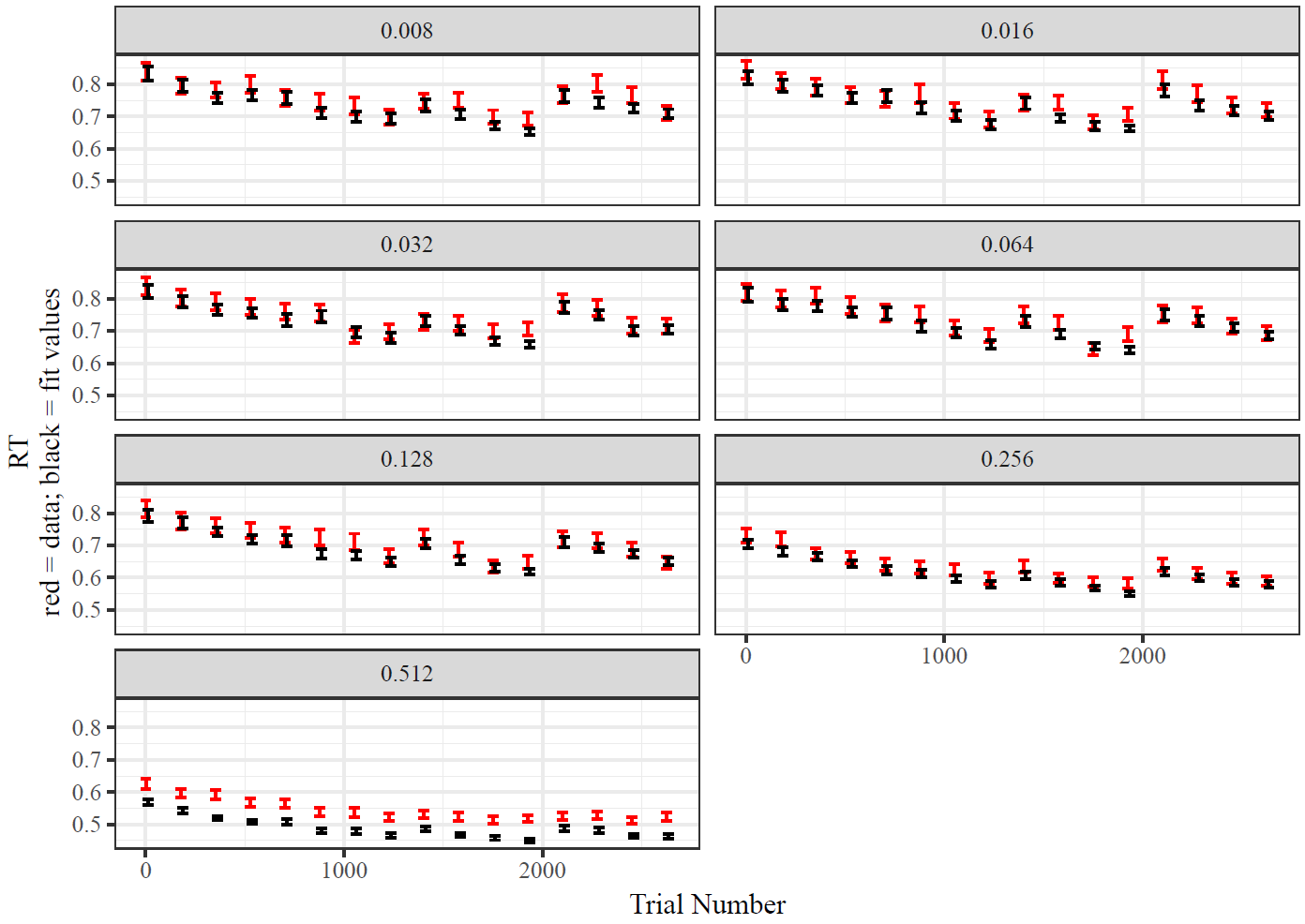
*

*Supplementary Figure 3.* ***Recovery of patterns of response times over time.*** *Separated by stimulus coherence level. Response times above the 97.5 and below the 2.5 percentile were first removed to mitigate the influence of extreme values, then raw accuracies and predicted values were each binned into blocks of 175 trials with 4 blocks per day for a total of 2800 trials over 7 coherences. Error bars indicate 95% confidence intervals.*


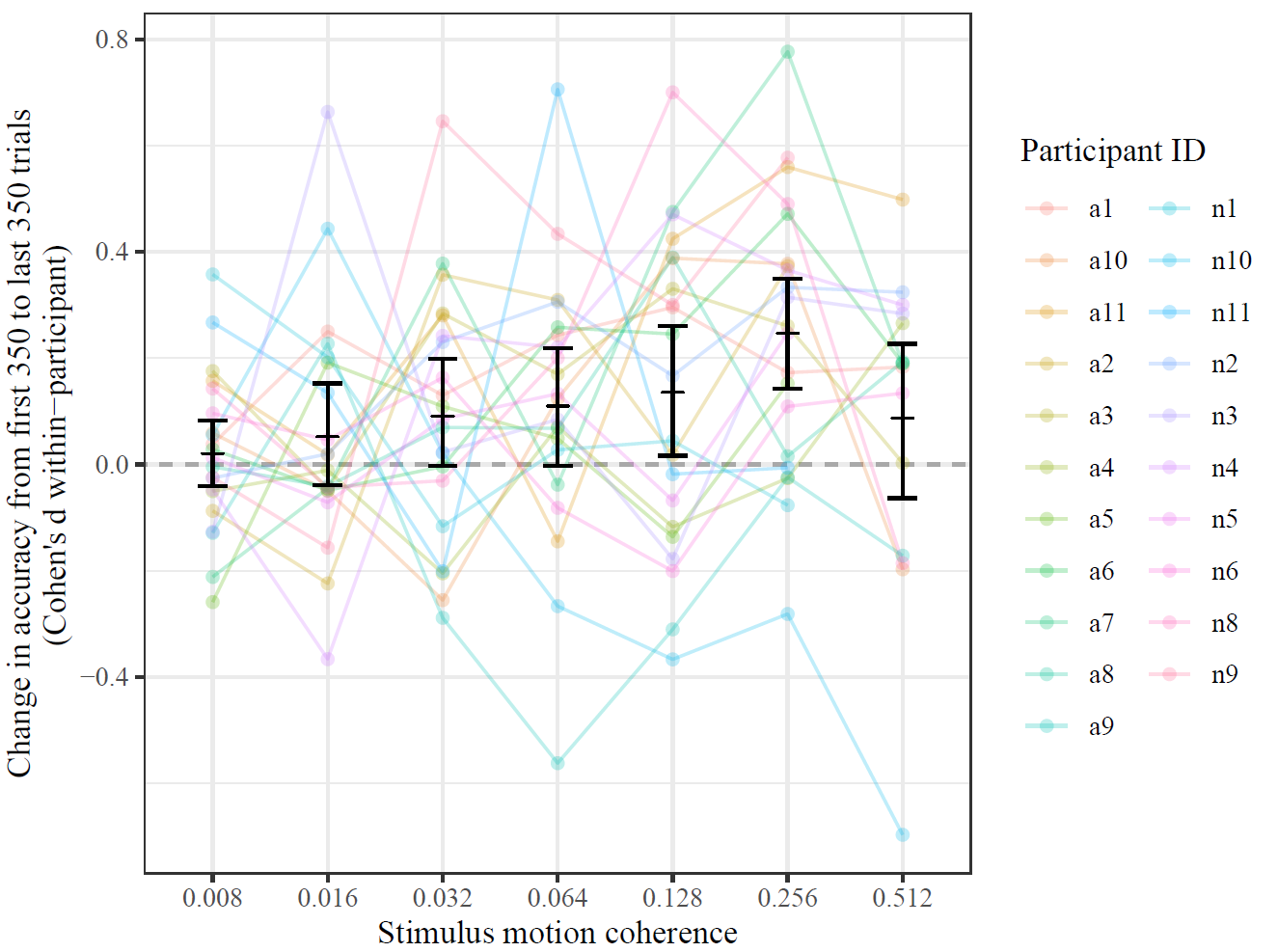


*Supplementary Figure 4.* ***Participant-level and group-level changes in accuracy****. Coherence levels .128 and .256 showed reliable increases in accuracy across participants, while all coherence levels showed numerical increases on average. The first half of the first day (350 trials) and the last half of the last day (350 trials) were compared for each participant and each coherence level. Yule correlations between binary accuracy and binary time (beginning=0, end=1) were calculated and converted into Cohen’s d for interpretation. Black lines indicate group-level means and bootstrapped 95% confidence intervals.*

*
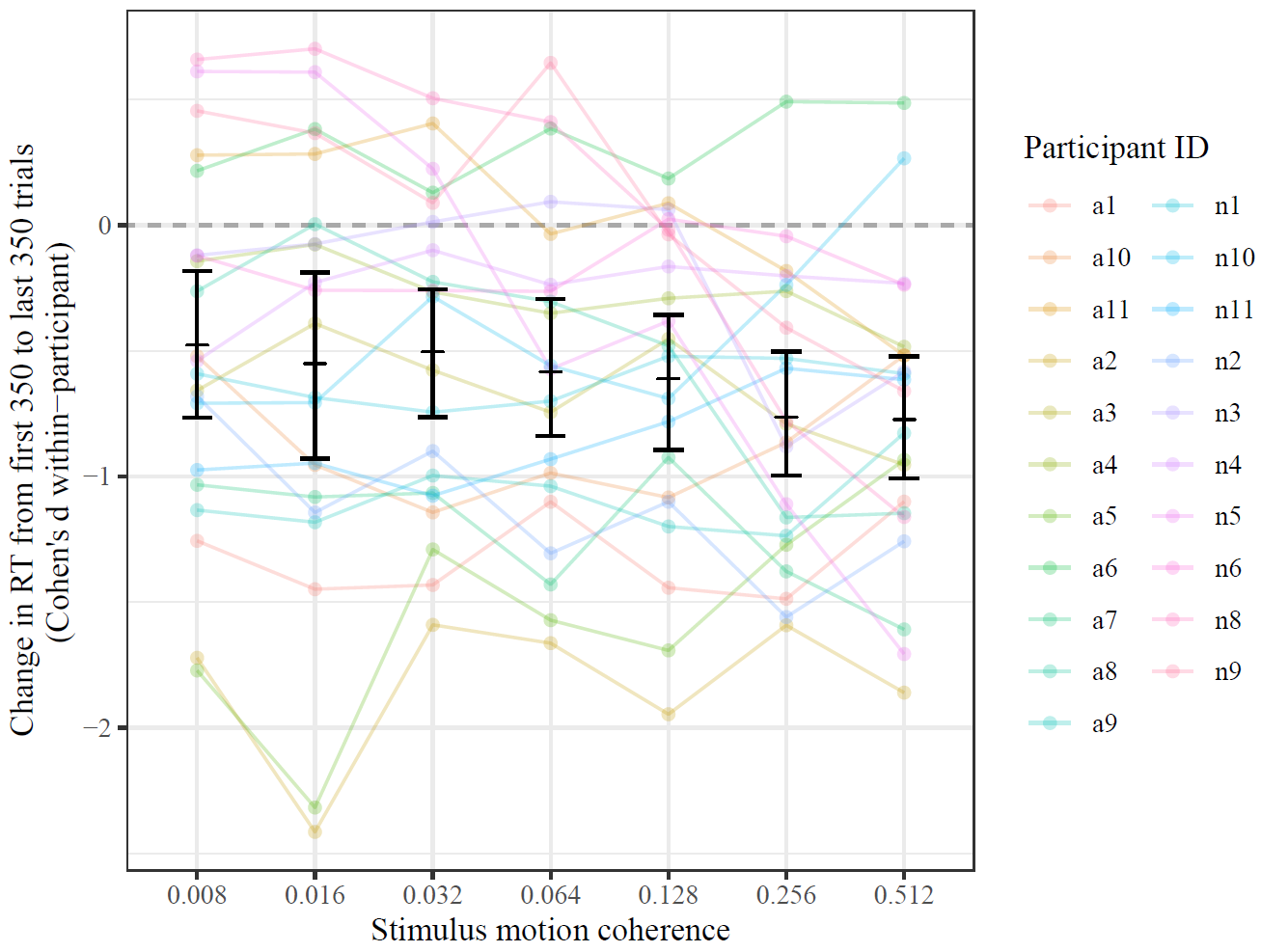
*

*Supplementary Figure 5.* ***Participant-level and group-level changes in response time****. All coherence levels showed reliable decreases in response time across participants. The first half of the first day (350 trials) and the last half of the last day (350 trials) were compared for each participant and each coherence level. Cohen’s d was calculated for the difference in the log-RT distributions between the beginning block and ending block. Black lines indicate group-level means and bootstrapped 95% confidence intervals.*

*Supplementary Table 1.* ***Fixed-effects estimates for the Constant DR, Constant RB model.*** *Estimate is posterior mean; Q2.5 and Q97.5 indicate the upper and lower 95% CI, respectively.*

|  | Estimate | Q2.5 | Q97.5 |
| --- | --- | --- | --- |
| Intercept | 0.596 | 0.518 | 0.675 |
| RB_Intercept | 0.459 | 0.377 | 0.54 |
| bias_Intercept | -0.264 | -0.309 | -0.22 |
| ndtOffset_Intercept | 0.223 | 0.196 | 0.249 |
| ndtOffset_isVGP | -0.042 | -0.09 | 0.008 |
| isVGP | 0.168 | -0.005 | 0.325 |
| RB_isVGP | -0.126 | -0.301 | 0.049 |
| bias_isVGP | -0.102 | -0.194 | -0.009 |

*Supplementary Table 2.* ***Fixed-effects estimates for the Continuous DR, Continuous RB model.*** *Estimate is posterior mean; Q2.5 and Q97.5 indicate the upper and lower 95% CI, respectively.*

|  | Estimate | Q2.5 | Q97.5 |
| --- | --- | --- | --- |
| bias_Intercept | -0.288 | -0.333 | -0.242 |
| DR_Asym_Intercept | 0.853 | 0.695 | 1.026 |
| DR_Asym_isVGP | 0.026 | -0.305 | 0.331 |
| DR_Start_Intercept | 0.413 | 0.317 | 0.507 |
| DR_Start_isVGP | 0.07 | -0.119 | 0.249 |
| DR_Rate_Intercept | 9.908 | 8.71 | 11.05 |
| DR_Rate_isVGP | -0.868 | -2.395 | 0.771 |
| RB_Start_Intercept | 0.592 | 0.462 | 0.721 |
| RB_Start_isVGP | -0.146 | -0.392 | 0.118 |
| RB_Rate_Intercept | 8.699 | 7.823 | 9.635 |
| RB_Rate_isVGP | 0.753 | -0.7 | 2.137 |
| RB_Asym_Intercept | 0.411 | 0.319 | 0.498 |
| RB_Asym_isVGP | -0.221 | -0.4 | -0.044 |
| ndtOffset_Intercept | 0.224 | 0.196 | 0.252 |
| ndtOffset_isVGP | -0.04 | -0.085 | 0.008 |
| bias_isVGP | -0.115 | -0.209 | -0.024 |

*Supplementary Table 3.* ***Fixed-effects estimates for the Continuous DR, Day-resetting RB model.*** *Estimate is posterior mean; Q2.5 and Q97.5 indicate the upper and lower 95% CI, respectively.*

|  | Estimate | Q2.5 | Q97.5 |
| --- | --- | --- | --- |
| bias_Intercept | -0.29 | -0.335 | -0.247 |
| DR_Asym_Intercept | 0.883 | 0.762 | 1.027 |
| DR_Asym_isVGP | 0.133 | -0.129 | 0.375 |
| DR_Start_Intercept | 0.383 | 0.286 | 0.478 |
| DR_Start_isVGP | 0.035 | -0.158 | 0.232 |
| DR_Rate_Intercept | 10.196 | 9.338 | 11.065 |
| DR_Rate_isVGP | -0.709 | -2.062 | 0.609 |
| RB_Asym_Intercept | 0.233 | -0.069 | 0.415 |
| RB_Asym_isVGP | 0.19 | -0.167 | 0.733 |
| RB_Rate_Intercept | 8.729 | 7.755 | 9.409 |
| RB_Rate_isVGP | -0.624 | -2.313 | 1.117 |
| RB_Start_Intercept | 0.506 | 0.393 | 0.62 |
| RB_Start_isVGP | -0.198 | -0.424 | 0.024 |
| ndtOffset_Intercept | 0.224 | 0.196 | 0.25 |
| ndtOffset_isVGP | -0.041 | -0.088 | 0.006 |
| bias_isVGP | -0.115 | -0.204 | -0.028 |

*Supplementary Table 4.* ***Fixed-effects estimates for the Flexible DR, Continuous RB model.*** *Estimate is posterior mean; Q2.5 and Q97.5 indicate the upper and lower 95% CI, respectively.*

|  | Estimate | Q2.5 | Q97.5 |
| --- | --- | --- | --- |
| bias_Intercept | -0.295 | -0.34 | -0.248 |
| DR_Asym_Intercept | 1.01 | 0.511 | 2.196 |
| DR_Asym_isVGP | -0.198 | -1.563 | 0.798 |
| DR_Asym_dayday2 | 0.719 | -0.091 | 2.145 |
| DR_Asym_dayday3 | 0.679 | 0.116 | 1.801 |
| DR_Asym_dayday4 | 0.653 | 0.231 | 1.579 |
| DR_Start_Intercept | 0.446 | 0.358 | 0.536 |
| DR_Start_isVGP | 0.174 | 0.002 | 0.342 |
| DR_Start_dayday2 | 0.093 | 0.002 | 0.187 |
| DR_Start_dayday3 | 0.161 | 0.052 | 0.273 |
| DR_Start_dayday4 | 0.147 | 0.052 | 0.241 |
| DR_Rate_Intercept | 9.193 | 8.455 | 9.441 |
| DR_Rate_isVGP | 0.021 | -1.543 | 1.615 |
| RB_Asym_Intercept | 0.407 | 0.317 | 0.495 |
| RB_Asym_isVGP | -0.214 | -0.383 | -0.046 |
| RB_Rate_Intercept | 8.547 | 7.645 | 9.504 |
| RB_Rate_isVGP | 0.822 | -0.578 | 2.181 |
| RB_Start_Intercept | 0.613 | 0.482 | 0.732 |
| RB_Start_isVGP | -0.143 | -0.394 | 0.101 |
| ndtOffset_Intercept | 0.224 | 0.196 | 0.252 |
| ndtOffset_isVGP | -0.04 | -0.085 | 0.01 |
| bias_isVGP | -0.119 | -0.211 | -0.035 |

*Supplementary Table 5.* ***Fixed-effects estimates for the Flexible DR, Flexible RB model.*** *Estimate is posterior mean; Q2.5 and Q97.5 indicate the upper and lower 95% CI, respectively.*

|  | Estimate | Q2.5 | Q97.5 |
| --- | --- | --- | --- |
| bias_Intercept | -0.299 | -0.342 | -0.257 |
| DR_Asym_Intercept | 1.947 | 0.644 | 4.624 |
| DR_Asym_isVGP | 0.159 | -2.096 | 2.845 |
| DR_Asym_dayday2 | 0.896 | -0.659 | 3.104 |
| DR_Asym_dayday3 | 0.817 | -0.744 | 2.902 |
| DR_Asym_dayday4 | 1.017 | -0.237 | 3.044 |
| DR_Start_Intercept | 0.448 | 0.364 | 0.534 |
| DR_Start_isVGP | 0.153 | -0.008 | 0.304 |
| DR_Start_dayday2 | 0.096 | 0.021 | 0.171 |
| DR_Start_dayday3 | 0.169 | 0.081 | 0.264 |
| DR_Start_dayday4 | 0.204 | 0.119 | 0.29 |
| DR_Rate_Intercept | 8.983 | 7.748 | 9.434 |
| DR_Rate_isVGP | 0.028 | -1.774 | 1.848 |
| RB_Asym_Intercept | 0.404 | 0.27 | 0.52 |
| RB_Asym_isVGP | 0.094 | -0.132 | 0.346 |
| RB_Asym_dayday2 | -0.125 | -0.3 | 0.033 |
| RB_Asym_dayday3 | -0.161 | -0.286 | -0.05 |
| RB_Asym_dayday4 | -0.047 | -0.162 | 0.068 |
| RB_Rate_Intercept | 8.473 | 7.706 | 9.24 |
| RB_Rate_isVGP | -0.046 | -1.31 | 1.227 |
| RB_Start_Intercept | 0.577 | 0.457 | 0.696 |
| RB_Start_isVGP | -0.166 | -0.427 | 0.082 |
| RB_Start_dayday2 | -0.094 | -0.171 | -0.021 |
| RB_Start_dayday3 | -0.096 | -0.182 | -0.012 |
| RB_Start_dayday4 | -0.085 | -0.174 | -0.001 |
| ndtOffset_Intercept | 0.224 | 0.196 | 0.252 |
| ndtOffset_isVGP | -0.042 | -0.086 | 0.006 |
| bias_isVGP | -0.117 | -0.201 | -0.034 |
